# Immunotherapy to reduce frequency of urinary tract infections in people with neurogenic bladder dysfunction; a pilot randomised, placebo-controlled trial

**DOI:** 10.1177/0269215520946065

**Published:** 2020-08-07

**Authors:** Derick T Wade, James Cooper, Nicholas Peckham, Maurizio Belci

**Affiliations:** 1 Oxford Institute of Nursing, Midwifery and Allied Health Research (OxINMAHR), Faculty of Health & Life Sciences, Oxford Brookes University, Oxford, UK; 2 NSIC Research Programme Manager, National Spinal Injury Centre (NSIC), Stoke Mandeville Hospital, Buckinghamshire Healthcare NHS Trust, Aylesbury, Bucks, UK; 3 Medical Statistician, Oxford Clinical Trials Research Unit, Centre for Statistics in Medicine, Nuffield Department of Orthopaedics, Rheumatology and Musculoskeletal Sciences, (NDORMS), University of Oxford, Botnar Research Centre, Headington, Oxford, UK; 4 Consultant in Spinal Cord Injuries, National Spinal Injury Centre (NSIC), Stoke Mandeville Hospital, Buckinghamshire Healthcare NHS Trust, Aylesbury, Bucks, UK

**Keywords:** Urinary infection, neurogenic bladder, immunotherapy, spinal cord injury, multiple sclerosis

## Abstract

**Objective:**

To establish the feasibility of a randomized, placebo-controlled trial to investigate the effect of a specific immunotherapy bacterial lysate OM-89 (Uro-Vaxom^®^) in reducing the frequency of urinary tract infections in people with neurogenic bladder dysfunction.

**Design:**

A parallel-group, double-blind, randomized, placebo-controlled trial.

**Setting:**

Patients at home, recruited through out-patient contact, social media and patient support groups.

**Subjects:**

People with a spinal cord injury, multiple sclerosis, transverse myelitis or cauda equina syndrome who had suffered three or more clinically diagnosed urinary tract infections treated with antibiotics over the preceding 12 months.

**Interventions:**

All participants took one capsule of oral OM-89 immunotherapy (6 mg) or matching Placebo (randomisation ratio 1:1), once daily in the morning for 3 months.

**Main measures:**

The primary outcome was occurrence of a symptomatic urinary tract infection treated with an antibiotic, assessed at 3 and 6 months. Feasibility measures included recruitment, retention and practical difficulties.

**Results:**

Of 115 patients screened, 49 were recruited, one withdrew before randomization, and 23 were allocated to the control group receiving matching placebo. Six participants, all in the control group, discontinued the intervention; all participants provided full data at both follow-up times. Over 6 months, 18/25 active group patients had 55 infections, and 18/23 control group patients had 47 infections. Most research and clinical procedures were practical, and acceptable to participants.

**Conclusion:**

It is feasible to undertake a larger trial. We recommend broader inclusion criteria to increase eligibility and generalizability.

## Introduction

Bladder control is disturbed in many neurological conditions, and dysfunction is often associated with an increased frequency of urinary tract infections which, in turn, can reduce quality of life, increase dependence on carers, and even precipitate hospital admission. Around two thirds of patients with neurogenic bladder disturbance have asymptomatic bacteriuria, and only 20% of these have a clinical urinary tract infection over the next year.^1^ Policies to reduce the risk of infection have little evidence to support them, are not very successful and may have other harmful effects, notably increasing the risk of bacterial resistance to antibiotics.^1,2^

The trial protocol has been published,^3^ and it details the previous evidence relating to immunotherapy using a specific immunotherapy bacterial lysate OM-89 (Uro-Vaxom^®^).^4,5^ Briefly, recurrent urinary tract infections are common in some people with neurogenic bladder dysfunction.^6,7^ There is no very effective preventative strategy. Immunotherapy offers the advantage of not inducing bacterial resistance to antibiotics. One randomized trial,^5^ published in 1990, suggested a benefit from immunotherapy that persisted after immunotherapy ended. A retrospective cohort study gave weak supportive evidence.^8^

We conducted a pilot, feasibility study in patients with a wider group of neurological disorders, in order to decide whether a large-scale clinical trial of an immunotherapy using oral OM-89 (Uro-Vaxom^®^) is possible, and to optimize trial design.

## Methods

This was a parallel-group, double-blind, randomized, placebo-controlled trial with blinded data analysis, which was undertaken between April 2018 and September 2019 and registered on ClinicalTrials.Gov in October 2015 (NCT02591901). The study was reviewed by the London (Harrow) Research Ethics Committee (ref: 15/LO/2069) and agreed on 1st March 2016. It was funded by the National Institute for Health Research (NIHR), through its Research for Patient Benefit funding stream (PB-PG-1013-32017). The data handling and analysis was undertaken by the Oxford Clinical Trials Research Unit (OCTRU). Its management was within the National Spinal Injuries Centre, Stoke Mandeville Hospital, and Buckinghamshire Healthcare NHS Trust were the sponsor. The active and placebo capsules of oral immunotherapy OM-89 were provided by the manufacturer, OM Pharma SA, free of charge. The full protocol has been published.^3^

The centres within this study were Stoke Mandeville Hospital, Wycombe Hospital, Amersham Hospital (part of Buckinghamshire Healthcare NHS Trust) and Oxford Centre for Enablement (part of Oxford University Hospitals NHS Foundation Trust). Participants were recruited from patients attending clinics or a day hospital, and through word of mouth, publicity on social media and patient support groups. Any patient who was interested saw a doctor or a nurse who gave further information and obtained consent.

Patients were eligible for the study if they had a neurological diagnosis (spinal cord injury, cauda equina syndrome, transverse myelitis, or multiple sclerosis) causing bladder dysfunction, **and** the neurological diagnosis had lasted more than 12 months, **and** had been clinically stable for 12 weeks or longer, **and** had experienced three or more clinically diagnosed urinary tract infections over the preceding 12 months. Patients who used urethral or supra-pubic catheters, intermittently or constantly, were eligible. Women of child-bearing age had to be willing to use contraceptive precautions for the duration of the treatment.

The exclusion criteria were:

- Having had a surgical procedure on the bladder, with the exception of a suprapubic catheter insertion,
- Having a known allergy to any of the contents of the OM-89 capsule,
- Being unwilling to take a capsule that included bovine gelatin.

The neurological diagnoses were all clinically based. The diagnosis of a urinary tract infection was also clinical: the diagnosis was accepted if the participant’s doctor had diagnosed, or confirmed the patient’s diagnosis of, a urinary tract infection **and** had prescribed or authorized the use of antibiotics as a treatment.

Once the patient’s eligibility had been confirmed, they were registered with the Oxford Clinical Trials Research Unit (OCTRU). Random allocation, on a 1:1 basis, was undertaken using a central computer-based system, Registration/Randomization and Management of Product (RRAMP), used by the unit. This system used a non-deterministic minimization algorithm with a probabilistic element of 0.8. The trials unit then contacted the hospital pharmacy, who stored pre-numbered, sealed packs containing active or placebo capsules, and stated which numbered pack should be given to the patient. The participant and the research team were blinded by treatment allocation. Only the trials unit (OCTRU) could identify which treatment any participant had.

The research nurse or doctor also collected baseline demographic and clinical data. Then the allocated drug package was given to the participant, by the site’s pharmacy, and the participant was asked to take one capsule each morning on an empty stomach for three consecutive months. They were asked to return any remaining capsules at the 12-week assessment point. Each participant was given a diary to record any urinary tract infections (date, symptomatology, antibiotic used).

Participants were told that they should continue with their usual way of responding to any suspected infection. For most people, this was contacting their general practitioner, but some participants had an emergency pack of antibiotics that they could start prior to making contact with their doctor. In the latter case, the episode was considered a urinary tract infection unless the doctor specifically gave an alternative diagnosis.

Study appointments were arranged after weeks 4 (telephone appointment), 12 (face to face) and 24 (face to face) to establish the occurrence of any infections, and check for adverse events or other concerns of the participant. The assessor could not know the patient’s allocated group.

The only clinical outcome data collected to assess the efficacy of OM-89 were:

- Occurrence of a urinary tract infection treated using an antibiotic. The date of the infection was taken from the patient’s diary. If a participant reported an infection by phone that was not in the diary, then the date given by phone was used.
- Urine samples were taken at weeks 12 and 24. They were analysed using routine microscopy, culture and antibiotic sensitivity testing.

In addition, participants were asked to note the first symptom and the major symptoms associated with an infection. These data are not part of this study report.

The primary feasibility questions were:

- How easy would it be to recruit 48 (the number aimed for) patients in 9 months?
- Would collecting and analysing urine samples collected at weeks 12 and 24 be practical, and
- Would the data add any useful information? Would collecting data on the occurrence of an infection be consistent between diaries and direct collection through phone or visit contacts?
- To test the suitability of collecting these data this way for a future larger trial.

The data analysis was undertaken blind. The sample size was not large enough for formal statistical analysis so the analysis was primarily descriptive.

## Results

**Figure 1.**
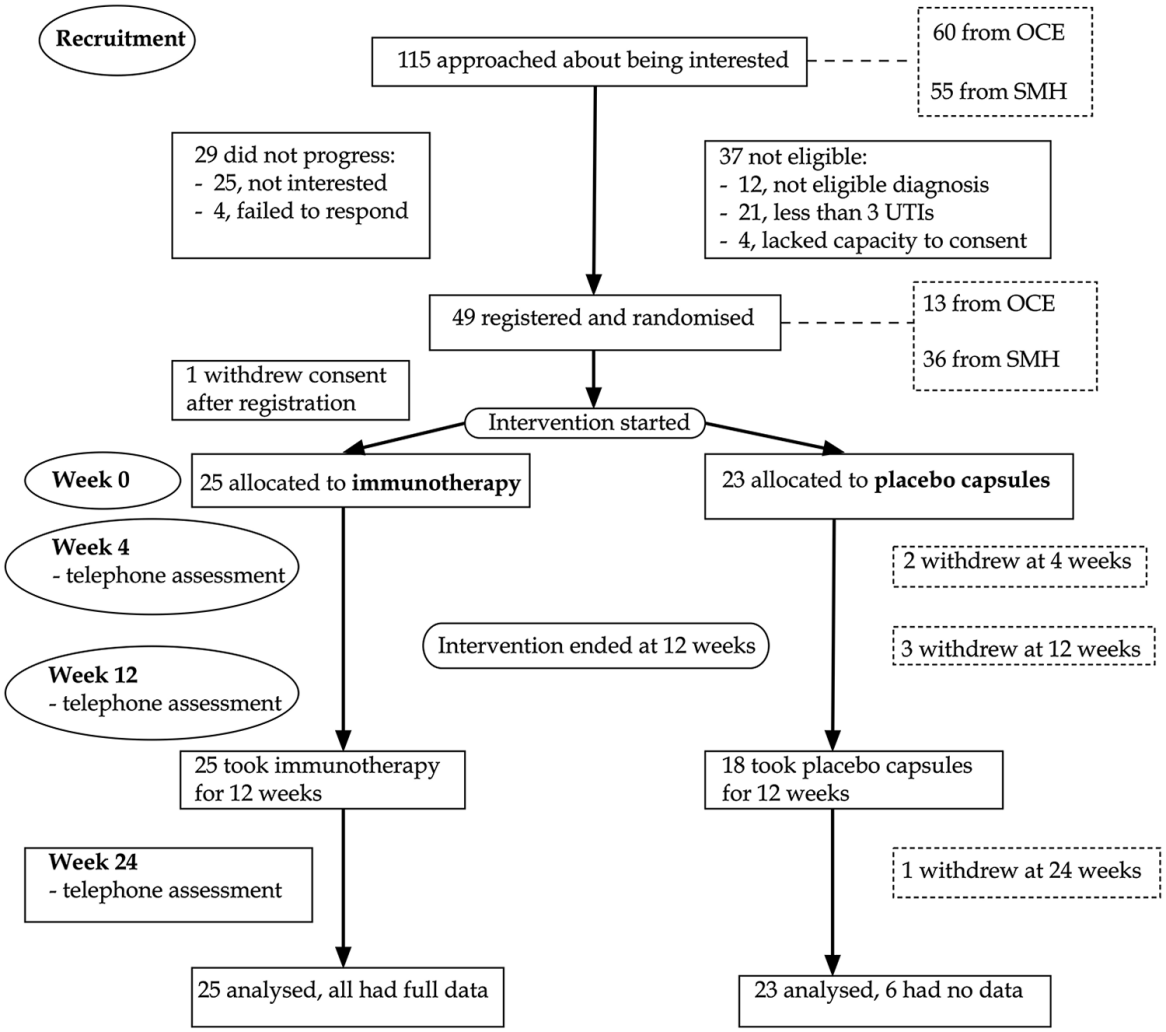
Trial flow diagram. OCE: Oxford Centre for Enablement; SMH: Stoke Mandeville Hospital; UTI: Urinary Track Infection.

A total of 115 patients were screened for eligibility, and the flow of patients is shown in the flow diagram (Figure 1). Five people, all in the placebo arm, stopped taking the capsules and six people, all in the placebo arm, withdrew during the trial - their outcome data were incomplete and not analyzed. No reasons were given. The demographic information is shown in Table 1.

**Table 1.** Study population: demographic and background data.

Item	Uro-Vaxom (Active) group (*n* = 25)	Placebo (control) group (*n* = 23)
**Age** – mean (SD) years	48.1 (11.8)	50.0 (11.2)
**Gender**: Male:female	12:13	8:15
**Site**: OCE:SMH	7:18	6:17
**Diagnosis**		
Spinal cord injury	17	15
Multiple sclerosis	8	8
**Catheter**		
Indwelling (SPC:urethral)	4:0	4:1
Intermittent	11	9
None	10	9
**Infections** over previous year		
3–5	16	9
6–8	6	8
9 or more	3	6
Total over year	138	156
**Antibiotic** emergency pack available	5	11

OCE: Oxford Centre for Enablement; SD: Standard Deviation; SMH: Stoke Mandeville Hospital; SPC: Suprapubic Catheter.

The number of infections in each group is shown in Table 2. There is no statistically significant difference between the groups. The agreement between the two methods of recording urinary tract infections – daily diary, or direct assessment through study appointments at 4, 12 and 24 weeks – is shown in Table 3. There was a close agreement.

**Table 2.** Urinary tract infections in study population.

Item	Active group *n* = 25	Placebo group *n* = 23
Number over 6 months		
Data missing	0	6
***Valid data***	*n* = 25	*n* = 17
Total number of infections	55	47
Mean (SE)	2.2 (0.46)	2.8 (0.54)
Mean (95% CIs)	2.2 (1.25, 3.15)	2.8 (1.62, 3.91)
Zero	7 (28%)	5 (29%)
1–4	13 (52%)	8 (47%)
5–8	5 (20%)	4 (24%)
Days to first infection	*n* = 18	*n* = 12
Range	4–179	2–62
Mean (SE)	58.1 (12.9)	30.8 (4.95)
Mean (95% CIs)	58.1 (30.71, 85.40)	30.8 (20.06, 41.63)

CI: 95% confidence interval; SE: Standard Error.

**Table 3.** Concordance in infection data between diary and telephone record.

Item	Active group *n* = 25	Placebo group *n* = 17
Week 4 record		
Diary	15	10
Study appointment	15	9
Difference	0	–1
Week 12 record		
Diary	17	21
Study appointment	20	22
Difference	+3	+1
Week 24 record		
Diary	23	19
Study appointment	18	20
Difference	–5	+1

No data on urine analysis are presented. Collection of urine samples was difficult, and inconsistent. In those samples analyzed between 75% and 83% were infected when collected. Adverse events reported are shown in Table 4.

**Table 4.** Adverse events recorded.

Item	Active group *n* = 25	Placebo group *n* = 23
**Adverse events**		
Urinary tract infection	60	52
Headache	8	19
Increased urine	8	9
Nausea	10	9
Sleep disorder	3	7
Back pain	2	8
Kidney pain	5	7
Diarrhoea	6	3
Flu-like symptoms	3	1
Heartburn	3	4
Rash	2	3
Flu	0	2
Vomiting	3	0
Vaginal soreness	0	2
Other	21	19
**Serious adverse events (all hospitalized)**		
Urinary infection	1	1
Pulmonary embolism	0	1
Pancreatitis	0	1
infected skin pressure ulcer	0	1

## Discussion

The main findings from this trial are as follows. It is quite feasible to recruit patients with a neurogenic bladder and recurrent urinary tract infections into a randomized trial of an oral immunotherapy aimed at reducing the rate of infection. Collecting data on how many infections a participant has and when is also feasible using daily diaries or telephone contacts. Attempting to study the bacteriology of the urine at fixed time points is not feasible, and is unlikely to be useful. Relaxing the entry criteria to recruit people with a single infection in the previous year would increase recruitment. Analyzing data relating to the time after the end of immunotherapy may be more likely to detect a difference, because it may take time for immunotherapy to work.

There is only one other published trial investigating immunotherapy in patients with neurogenic bladder dysfunction and recurrent urinary tract infections, and that included 70 participants.^5^ Comparison is difficult as it was a cross-over design, but it was notable in that trial that the effect was greater in the 3 months after the treatment period. This is to be expected, if the therapy induces a bodily response specifically against one bacterium. This current study was underpowered to detect any effect, but we note that the delay to first infection was longer in the active group.

There is, however, one small randomized trial (*n* = 26) of inoculation of Escherichia coli into the bladder, and one interpretation of the data is that the rate of subsequent infection was reduced.^8^ This trial also relied on the same definition of urinary tract infection as in our trial. Their hypotheses appeared to be that subclinical infection with a ‘benign strain’ reduced the opportunity for other bacteria to cause infection. An alternative hypothesis could be that subclinical infection induces a local immune response in the urinary tract.

The mechanism by which this may reduce infections has not been established, but one possibility is that it induces local immunity in the lower urinary tract. Therefore, in any future trial, we would recommend analyzing separately infections over the first 3 months when, if it is effective, immunity is being established, and infections occurring after 3 months when immunity should be established. We would also recommend analyzing time from initiating therapy to first urinary tract infection. Last, it would also seem sensible to analyze time to first infection from the end of immunotherapy (i.e. ‘resetting the clock’), because the treatment may have a cumulative effect, with little effect in the early stages which would introduce noise in the data.

One obvious criticism of the trial is the uncertainty associated with the diagnosis of a urinary tract infection. Unfortunately, there is in fact no good way to make the diagnosis.^1^ It was obvious even before starting the trial that it would be impossible to obtain a urine sample whenever an infection was suspected before starting antibiotics. Equally importantly, urine microscopy and culture is not especially valid.

We decided that, in day-to-day clinical practice the diagnosis is made clinically, and it was the rate of this clinical diagnosis we wished to reduce, so relying on a pragmatic, clinical definition was quite appropriate. Others have used the same approach: ‘Self-reported UTIs were considered clinically relevant, because patients present for UTI treatment when they experience symptoms’^8^; and ‘The decision of antibiotic therapy was made by the patient based only on subjective symptoms’.^9^

Another criticism could be a reliance on self-report of diagnoses and events. We did not cross-confirm the diagnosis of infection against medical records, which would have required contacting the general practitioner. This would have been impractical, and would not give uniform data. It was reassuring that the agreement between two methods was good, suggesting the numbers are reasonably accurate.

A second weakness is the loss of valid data which affected the placebo group. There was no obvious reason. This study was not intended to show effectiveness, and no bias was introduced.

This trial has shown that further research is possible. A systematic review of studies in healthy people^4^ and two studies in people with neurogenic bladder dysfunction provide sufficient evidence to warrant a larger trial. The data from this study suggest that 350 people would be needed to determine whether oral OM-89 immunotherapy benefits people with neurogenic bladder dysfunction and recurrent urinary tract infection.

**Clinical messages**

- Undertaking a trial of an immunotherapy using oral OM-89 immunotherapy to reduce the risk of recurrent urinary tract infections in people with neurogenic bladder dysfunction is feasible.
- The results are consistent with previous research and support undertaking a fully-powered study involving 350 participants.
